# No need to panic over the release of ‘treated water’ containing tritium from Fukushima Daiichi Nuclear Power Station

**DOI:** 10.1093/qjmed/hcad211

**Published:** 2023-09-21

**Authors:** I Amir, N Ito, M Tsubokura

**Affiliations:** Department of Radiation Health Management, Fukushima Medical University School of Medicine, Hikarigaoka-1, Fukushima City, Fukushima 960-1295, Japan; Department of Radiation Health Management, Fukushima Medical University School of Medicine, Hikarigaoka-1, Fukushima City, Fukushima 960-1295, Japan; Department of Radiation Health Management, Fukushima Medical University School of Medicine, Hikarigaoka-1, Fukushima City, Fukushima 960-1295, Japan

On 24 August, Tokyo Electric Power Company started releasing water containing tritium, called ‘treated water’, processed by Advanced Liquid Processing System. Some have panicked about the health effect of radiation from ‘treated water’ via the sea-food chain or water intake, which could lead to unfounded rumors without any basis in scientific facts. Since the ‘treated water’ has been diluted by sea water, its radiation level from tritium is one-fortieth the safety level under the criteria of the Japanese government and one-seventh the drinking water level designated by World Health Organization.^1^

Looking back to 12 years ago, after the accident of Fukushima Daiichi Nuclear Power Station (FDNPS), people feared the health effects of radiation from the radioactive material. However, according to the Fukushima Health Management Survey, there has been no direct health effects from radiation exposure.^2,3^ Rather, secondary health effects due to forced evacuation after the FDNPS accident are higher than expected.^4^ Based on these facts, United Nations Scientific Committee on the Effects of Atomic Radiation (UNSCEAR) has stated that ‘future radiation-associated health effects are unlikely to be discernible’ in the 2020/2021 report.^5^

For this occasion, because the ‘treated water’ released into ocean is scientifically safe under both criteria, adverse health effects of radiation are not likely to occur. As an academic and a medical organization located in Fukushima, our mission is to present scientific facts clearly to address the unfounded rumors about the ‘treated water’ based on non-scientific beliefs and to strengthen the risk communication on ‘treated water’.
